# Accidental Intravenous Administration of Oral Bacillus clausii Probiotic (Enterogermina) in a Toddler: A Case Report

**DOI:** 10.7759/cureus.107657

**Published:** 2026-04-24

**Authors:** Vidya Puttagunta, Sathya Seelan, Abdelhameed Elmesery

**Affiliations:** 1 Department of Pediatrics, NMC Royal Hospital, Abu Dhabi, ARE; 2 Department of Pediatrics, Prime Hospital, Dubai, ARE

**Keywords:** bacillus clausii, intravenous injections, medication errors, pediatric, preschooler, probiotics, toddler

## Abstract

Acute diarrheal illness is a common cause of hospitalization in toddlers, and probiotics such as oral *Bacillus clausii* suspension (Enterogermina) are frequently prescribed to restore gut flora. This report describes the first documented case in the United Arab Emirates of accidental intravenous administration of oral *Bacillus clausii* probiotic in a 17-month-old toddler. The previously healthy child presented with rotavirus-positive gastroenteritis and moderate dehydration. On the day of admission, 3 mL of Enterogermina containing approximately 2 billion spores was inadvertently given intravenously instead of orally. Within two hours, the toddler developed rigors and high-grade fever. He was immediately transferred to the pediatric intensive care unit, where empirical intravenous piperacillin-tazobactam and teicoplanin were started. Inflammatory markers rose sharply, but blood cultures remained sterile. The child became afebrile within 48 hours, markers normalized rapidly, and he was discharged in excellent condition after seven days of intravenous antibiotics with no long-term sequelae. This case demonstrates that even generally safe oral probiotics can trigger a significant but transient systemic inflammatory response when administered intravenously. Prompt recognition, intensive monitoring, and broad-spectrum antibiotics prevented serious complications. It highlights the urgent need for strict medication safety protocols, staff training, and clearer product labeling to avoid such preventable errors in pediatric practice.

## Introduction

Acute diarrheal illnesses remain a leading cause of morbidity in young children worldwide, particularly in resource-limited settings, and rank as the second most common cause of childhood mortality after respiratory infections when severe dehydration develops [1]. Rotavirus is one of the predominant pathogens responsible for severe pediatric gastroenteritis, often disrupting the normal gut microbiota by depleting beneficial bacteria such as *Lactobacillus*, *Bifidobacterium*, and *Enterococcus* species [2]. This dysbiosis prolongs the duration of diarrhea, impairs intestinal barrier function, and weakens local immune responses.

Probiotics have gained widespread acceptance as adjunctive therapy in acute gastroenteritis to restore microbial balance, shorten illness duration, and reduce hospital stay. Among them, *Bacillus clausii*, a spore-forming, Gram-positive, aerobic bacterium, stands out due to its high resistance to heat, gastric acid, and many antibiotics [3]. The commercial preparation Enterogermina contains four antibiotic-resistant strains (O/C, N/R, SIN, and T) of *Bacillus clausii *and is commonly prescribed to hospitalized children with diarrhea [4]. Meta-analyses of randomized trials have demonstrated that *Bacillus clausii *can significantly reduce the duration of diarrhea by approximately nine hours and the duration of hospitalization by nearly one day in children with acute gastroenteritis, while also showing immunomodulatory effects that may decrease recurrent respiratory infections [5].

Despite its excellent safety profile when administered orally, medication errors involving incorrect route of administration can lead to serious consequences. Accidental intravenous (IV) injection of oral probiotic suspensions has been documented in a limited number of cases globally [6,7]. Reported outcomes range from transient inflammatory responses and fever to bacteremia, prolonged sepsis requiring extended antibiotic therapy, anaphylactic reactions, hypotension, and respiratory distress. The manufacturer of Enterogermina (Sanofi, Paris, France) has been notified of at least 18 such erroneous IV administrations, highlighting that even generally safe spore-forming probiotics can trigger systemic effects when introduced directly into the bloodstream.

This case report describes the clinical course, laboratory findings, management, and outcome of a 17-month-old toddler who accidentally received 3 mL of oral Enterogermina intravenously during treatment for rotavirus gastroenteritis. The aims of this report are to highlight the potential inflammatory consequences of this rare medication error, emphasize the importance of strict adherence to administration protocols, and demonstrate that prompt recognition, intensive monitoring, and empirical broad-spectrum antibiotic therapy can lead to a favorable outcome without long-term morbidity. To our knowledge, this represents the first documented case of accidental IV *Bacillus clausii* administration in the United Arab Emirates (UAE).

## Case presentation

A previously healthy 17-month-old male toddler (weight: 10.2 kg) presented to the Pediatric Emergency Department of a tertiary care hospital in the UAE in March 2025, with a two-day history of non-bilious, non-projectile vomiting (approximately five to six episodes/day), watery non-bloody diarrhea (six to eight loose stools/day), poor oral intake, and reduced urine output. He had received all age-appropriate immunizations as per the UAE national immunization schedule, including the rotavirus vaccine series, and had no significant past medical or surgical history. There was no history of recent travel, sick contacts, or antibiotic use.

On physical examination, the child appeared lethargic and irritable. Vital signs on arrival showed low-grade fever, tachycardia, mild tachypnea, low-normal blood pressure, and normal oxygen saturation on room air. No history of high-grade or fluctuating fever was reported during the preceding two days. Clinical assessment indicated moderate dehydration (estimated 7-8% fluid deficit), evidenced by dry mucous membranes, sunken eyes, decreased skin turgor with prolonged capillary refill time, and absence of tears. The abdomen was mildly distended with increased bowel sounds; no organomegaly or peritoneal signs were elicited.

Initial laboratory investigations revealed a full blood count within normal limits, with a normal total leukocyte count and a balanced differential distribution, along with normal hemoglobin and platelet levels. Serum electrolytes were within normal ranges, including sodium, potassium, and chloride. Renal function parameters were also within normal limits. A mild elevation in C-reactive protein (CRP) was noted. Stool rapid antigen testing was positive for rotavirus. A provisional diagnosis of acute rotavirus gastroenteritis with moderate dehydration was established, and the child was admitted to the general pediatric ward for IV fluid resuscitation and supportive care (Table 1).

**Table 1:** Serial laboratory parameters, clinical events, and antibiotic therapy over the course of hospital admission CRP: C-reactive protein; PCT: Procalcitonin; Temp: Core body temperature; IV: Intravenous administration route; Pip-Tazo: Piperacillin-Tazobactam; PICU: Pediatric intensive care unit; Echo: Echocardiography (cardiac ultrasound).

Day	CRP (mg/L)		PCT (ng/mL)		Temperature (^0^C)		Key clinical events	Antibiotic therapy
	Value	Reference	Value	Reference	Value	Reference		
Day 1	7.1	<5	—	<0.5	38.0	36.1–37.2	IV error (10:50) PICU admission	IV Pip-Tazo + Teicoplamin
Day 2	32.4	<5	23.25	<0.5	39.0	36.1–37.2	Antibiotics commenced (IV Pip-Tazo + Teicoplanin)	IV Pip-Tazo + Teicoplanin
Day 3	7.1	<5	—	<0.5	38.8	36.1–37.2	—	IV Pip-Tazo + Teicoplanin
Day 4	4.7	<5	3.34	<0.5	37.2	36.1–37.2	Patient afebrile	IV Pip-Tazo + Teicoplanin
Day 7	<1	<5	<1	<0.5	36.8	36.1–37.2	PICU discharge	Oral Cefixime
Day 8	<1	<5	<1	<0.5	36.8	36.1–37.2	—	Oral Cefixime
Day 15	<1	<5	<1	<0.5	36.8	36.1–37.2	Echocardiogram normal and discharge home	Oral Cefixime

Medication error and immediate response

On the day of admission, a nurse inadvertently administered 3 mL of Enterogermina oral suspension (Sanofi; each 5 mL vial containing approximately 2 × 10⁹ spores of *Bacillus clausii*, strains O/C, N/R, SIN, and T) intravenously via a peripheral IV cannula, instead of the intended oral route. Since the preparation was intended for oral administration and not for IV use, it was not handled under sterile conditions prior to the inadvertent IV administration. The error was identified and reported immediately in accordance with hospital incident reporting protocols. The IV line was promptly discontinued and a new peripheral access was established. Approximately two hours following the inadvertent IV administration, the child developed rigors and a high-grade fever. There was no associated hypotension, altered consciousness, or new focal neurological signs. Given the clinical concern for iatrogenic bacteremia or systemic inflammatory response secondary to IV inoculation of bacterial spores, the child was promptly transferred to the pediatric intensive care unit (PICU) for hemodynamic monitoring and close observation.

PICU course and investigations

Two sets of peripheral blood cultures were drawn prior to antibiotic initiation. Empirical broad-spectrum IV antibiotic therapy was commenced, comprising piperacillin-tazobactam 100 mg/kg/day in three divided doses (targeting gram-negative organisms and anaerobes) and teicoplanin 10 mg/kg once daily (targeting gram-positive organisms, including potential *Bacillus* species). IV fluid resuscitation and antipyretics were continued.

On day two, the child remained febrile with persistent irritability. Serial inflammatory markers demonstrated a marked acute-phase response, with significantly elevated CRP and a marked rise in procalcitonin (PCT) levels. Renal function, hepatic transaminases, and coagulation profile remained within normal limits. The child was hemodynamically stable throughout, with no requirement for vasopressor support or supplemental oxygen.

A progressive clinical and biochemical improvement was observed over the subsequent days (Table 1). The child became afebrile by day three. By day four, both CRP and PCT levels had declined significantly, correlating with clinical improvement. All serial blood cultures obtained during admission showed no bacterial growth after incubation. By day seven, inflammatory markers had returned to normal levels. The total leukocyte count remained within normal limits throughout, with no band forms observed. Although repeat stool testing to document viral clearance was not performed, the diarrheal symptoms resolved completely by day three of hospitalization, suggesting clinical recovery from rotavirus infection.

Cardiac evaluation

Given the potential risk of hematogenous seeding of cardiac valves following IV introduction of bacterial spores, a transthoracic echocardiogram was performed on day eight. It demonstrated a structurally normal heart with preserved biventricular systolic function, no valvular abnormalities, and no evidence of vegetations or endocarditis.

Antibiotic course and discharge

IV antibiotics were continued for a total of seven days. On day eight, antibiotic therapy was de-escalated to oral cefixime 8 mg/kg once daily to complete a total 15-day antibiotic course. The rotavirus gastroenteritis resolved completely during the admission, with normalization of stool frequency and consistency. The child was discharged home on day nine in satisfactory condition, tolerating full oral feeds, afebrile, and hemodynamically stable. At the follow-up outpatient visit on day 15 post the incident, the child was well, with normal appetite, activity level, and developmental milestones. No residual symptoms or sequelae attributable to the medication error were identified.

## Discussion

Accidental IV administration of oral *Bacillus clausii* probiotic (Enterogermina) remains a rare but clinically significant medication error. To date, only four detailed cases have been published in the English literature, all involving adults or older children [6-9]. The present report describes the first documented pediatric case in the UAE and one of the very few cases reported in a toddler worldwide. In marked contrast to previous reports, our previously healthy 17-month-old child with rotavirus gastroenteritis developed only a transient systemic inflammatory response. The toddler experienced high-grade fever with peak elevations in CRP and procalcitonin, yet blood cultures remained sterile throughout. Inflammatory markers normalized rapidly within days, and the child achieved full clinical recovery after seven days of IV antibiotics followed by oral step-down therapy. This benign course differs sharply from outcomes observed in older or more vulnerable patients.

Monnerat et al. [6] reported the index adult case of culture-positive *Bacillus clausii* septicemia that persisted for five months after inadvertent IV injection of 2 billion spores. Mehta et al. [7] described a 14-year-old post-surgical patient who developed marked leukocytosis with positive blood cultures and required nine days of parenteral antibiotics. In immunocompromised hosts, the consequences were far more protracted: Bilbao et al. [8] documented persistent bacteremia lasting 175 days in a 64-year-old man with chemorefractory lymphoma, complicated by infusion-site thrombophlebitis that necessitated surgical excision. Similarly, Walunj et al. [9] reported prolonged *Bacillus clausii *bacteremia for 52 days in a 46-year-old woman with end-stage renal disease awaiting transplantation, which cleared only after administration of long-acting dalbavancin.

The favorable outcome in our toddler is most likely explained by intact host immunity, the absence of indwelling central venous catheters, and immediate aggressive management. Prompt transfer to the PICU within hours of the error, combined with empirical piperacillin-tazobactam and teicoplanin, both highly active against *Bacillus clausii, *prevented sustained bacteremia [3]. *Bacillus clausii* spores can survive in the bloodstream and trigger a brisk inflammatory cascade through pathogen-associated molecular patterns even without active replication, which accounts for the striking elevation in inflammatory markers despite repeatedly negative blood cultures.

Although *Bacillus clausii* is generally considered safe when administered orally, probiotics are not risk-free for all patients. Bezirtzoglou and Stavropoulou [10] highlighted the critical role of intestinal microflora in the immunological development of newborns and young children, demonstrating that probiotics can beneficially modulate gut colonization, inhibit pathogenic organisms, and support immune maturation in this age group. However, Katkowska et al. [11] reviewed multiple cases of infections, including bacteremia, directly attributable to probiotic strains and emphasized that supplementation should be approached with caution, particularly in hospitalized or immunocompromised individuals. Similarly, Gul and Durante-Mangoni [12], in their comprehensive review, highlighted that while probiotics confer gastrointestinal, immune, and metabolic benefits, certain populations, including hospitalized, critically ill, and immunocompromised patients, are at an increased risk of serious adverse events such as sepsis and opportunistic infections. This dual nature underscores the potential for significant harm when an oral probiotic suspension such as Enterogermina is inadvertently administered intravenously, as occurred in the present case.

This case carries important clinical implications. Even probiotics regarded as generally safe can produce significant harm when administered by the wrong route, particularly in pediatric settings where oral suspensions are frequently prepared alongside IV medications. Pediatric patients are especially vulnerable during busy shifts or when medication storage and preparation areas are not strictly segregated.

Future recommendations should focus on prevention at multiple levels. Manufacturers should redesign Enterogermina packaging with prominent "ORAL USE ONLY" warnings, tamper-evident oral syringes, or vial colors incompatible with IV connectors. Hospitals must implement mandatory barcode scanning linked to route verification, separate storage of oral probiotics, and double-independent checks for high-alert medications in pediatric wards. Protocols for suspected errors should mandate immediate PICU admission, paired blood cultures from separate sites, echocardiography if bacteremia persists beyond 48 hours, and broad-spectrum antibiotic coverage until final culture results are available. In immunocompromised patients, early infectious-disease consultation and consideration of prolonged therapy are essential. Regular medication-safety training and simulation drills for nurses and residents should specifically address this type of error.

## Conclusions

This is the first reported case in the UAE of accidental IV administration of oral *Bacillus clausii *probiotic (Enterogermina) in a toddler. The child developed only a transient inflammatory response, characterized by fever and elevated inflammatory markers, but without bacteremia or long-term complications. Prompt pediatric intensive care unit admission and empirical broad-spectrum antibiotic therapy led to rapid and complete recovery. This case emphasizes that even generally safe oral probiotics can cause significant harm when administered intravenously, and highlights the critical need for strict medication safety protocols and staff training to prevent such errors in pediatric practice.
